# Post-tonsillectomy Hemorrhage: A Seven-year Retrospective Study

**DOI:** 10.22038/ijorl.2021.54962.2882

**Published:** 2021-09

**Authors:** Afsaneh Mohammadpour-Maleki, Bashir Rasoulian

**Affiliations:** 1 *Sinus and Surgical Endoscopic Research Center, Mashhad University of Medical Sciences, Mashhad, Iran.*

**Keywords:** Complication, Hemorrhage, Tonsillectomy

## Abstract

**Introduction:**

Post-tonsillectomy hemorrhage (PTH) is a serious complication that sometimes requires immediate surgical interventions. The present study aimed to assess the association between patients’ age, the time of onset of PTH, and the need for surgery to control bleeding.

**Materials and Methods:**

All patients with PTH were retrospectively admitted to two tertiary hospitals in Mashhad, during 2012-2019. Hospital records were investigated to select eligible cases and retrieve their characteristics such as demographics, source and time of bleeding, and type of intervention. Chi-square, independent samples T-test, and binary logistic regression were used as research tools.

**Results:**

A total of 227 patients with PTH and a mean age of 14.99±10.34 years were studied, of whom 128 (56.4%) were male and 63 (27.8%) required surgery to control PTH. The mean onset of PTH was 8.14±3.47 days after the surgery and in 59 cases (26.5%) was the seventh day. Those patients aged 6 years or older in whom PTH occurred during the first postoperative week were significantly more likely to need surgery to control it (P= 0.034). Adult (OR= 4.032, 95%CI= 1.932-8.414, P<0.001), bleeding from both tonsils (OR= 2.380, 95%CI= 1.032-5.487, P= 0.042), and receiving blood transfusion (OR= 7.934, 95%CI= 2.003-31.422, P= 0.002) were independent predictors of the need for surgical treatment to control PTH.

**Conclusion:**

PTH within the first postoperative week in patients older than 6 years, adults, bleeding from both tonsils, and receiving a blood transfusion is recommended to be considered as a potential predictor of the need for surgery.

## Introduction

Tonsillectomy is one of the most common otolaryngological surgical procedures. In the United States, 289,000 ambulatory procedures are performed annually. Complications of tonsillectomy may include sore throat, postoperative nausea, and vomiting, dehydration, otalgia, Eustachian tube dysfunction, delayed feeding, velopharyngeal incompetence, pulmonary edema, bleeding, and rarely death (1-4).

Post-tonsillectomy hemorrhage (PTH) is one of the most serious complications that may occur at any time in the postoperative period. PTH is often divided into primary (within 24 hours of surgery) and secondary (24 hours after surgery) in the literature. About 0.2% to 2.2% and 0.1% to 3.3% of patients experience primary and secondary PTH, respectively. Patients might be hospitalized to control PTH or surgery (1,2,5-7). Therefore, identifying patients who need surgical treatment is more important to ensure timely and proper management.

In this study, PTH and associated features were retrospectively reviewed in a population with a wide age range. The present study aimed to assess the association between patients’ age, time of PTH, and the need for surgery to control bleeding.

## Materials and Methods

The study population in this retrospective study were all patients with PTH who were referred to Imam Reza and Ghaem hospitals in Mashhad, Iran from January 2012 to September 2019. The present study was approved by the Ethics committee of Mashhad University of Medical Sciences, Mashhad, Iran (approval code: IR.MUMS.MEDICAL.REC.1398.679). Patient consent was waived by the institutional review board and the ethics committee due to the retrospective nature of the study.

All patients who underwent tonsillectomy or adenotonsillectomy and presented with PTH were retrospectively included in the study. All patients were operated in the same conditions with the cold knife and bipolar cauterization techniques. The patients who had undergone the initial surgery at a different hospital had incomplete information profiles, or were referred from another center were excluded from the study.Hospital records were searched for diagnostic code T81.0 of the International Classification of Diseases, Tenth Revision (ICD-10), which indicates post-operative bleeding, hematoma, or seroma complications. After a primary list of patients was acquired, all medical charts were manually checked to select those which met the inclusion criteria. Patient demographics, bleeding time, and type of intervention (non-operative or operative) were extracted.

Statistical analysis

Statistical analyses were performed using SPSS software (version 16). For dichotomous variables, descriptive analysis was performed by calculating the relative frequency of each category. Mean and standard deviation was calculated to present data for continuous variables. A Chi-square test was used to compare the frequency of postoperative bleeding. Independent samples t-test was performed to compare the days of postoperative bleeding. A binary logistic regression model was used to evaluate the association of different factors with the need for surgery. P-value less than 0.05 was considered statistically significant in all tests.

## Results

A total of 227 patients with PTH and a mean age of 14.99±10.34 years (range 2-53 years) were studied during seven years, of whom 128 (56.4%) were male and 63 (27.8%) required surgery to control PTH.

Also, 164 (72.2%) recovered without any surgical intervention, while 63 (27.8%) underwent surgery to control PTH. Of these 63 patients, two (3.2%) had a second hemorrhage and underwent another surgery to control re-bleeding.

**Fig 1:**
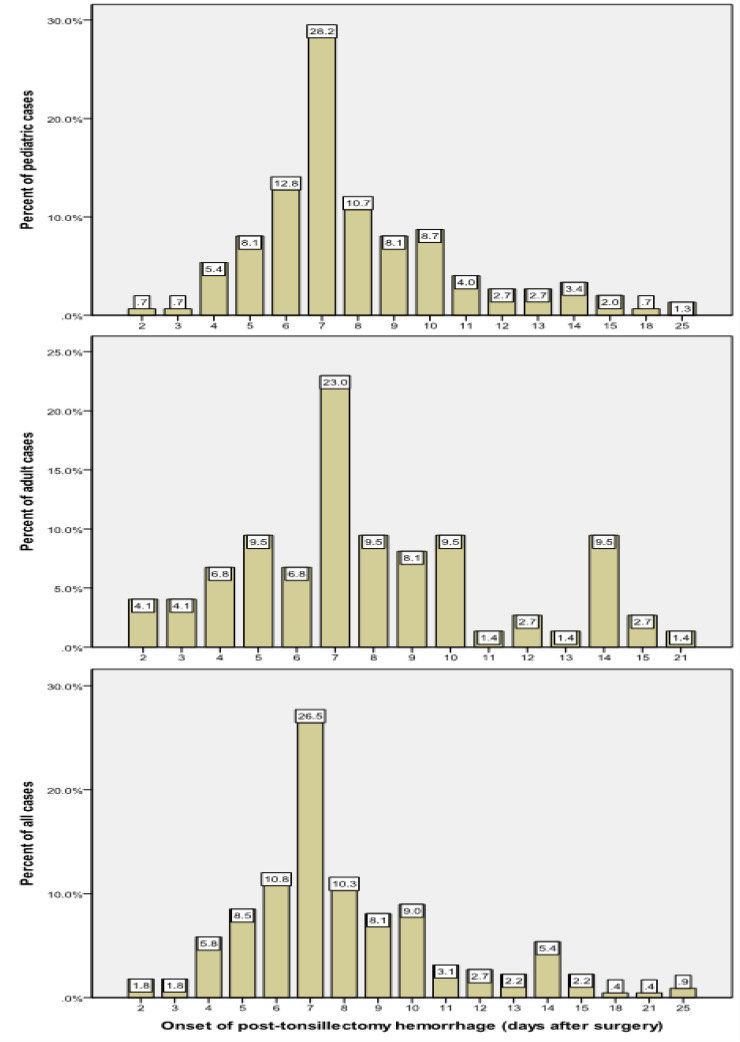
Distribution of onset of post-tonsillectomy hemorrhage

In total, 12 (5.3%) patients had received an intraoperative blood transfusion. On average, PTH occurred 8.14±3.47 days after the surgery (range 2-25 days). The largest group of patients (N= 59, 26.5%) had PTH 7 days after the tonsillectomy surgery and most patients (N= 121, 55.3%) experienced bleeding within one week of operation (Fig. 1).

Table 1 compares demographic and clinical information between patients who underwent surgery and those who received non-surgical treatments for PTH. 

**Table 1:** Demographic and clinical characteristics of patients in the surgical and non-surgical treatment groups

Variable		Treatment		Total	P*
		Surgical(N=63)	Non-surgical(N=164)		
**Gender**	Male Female	34 (26.6%) 29 (29.3%)	94 (73.4%) 70 (70.7%)	128 99	0.649
**Age (years)**	<18 ≥18	33 (21.9%) 30 (39.5%)	118 (78.1%) 46 (60.5%)	151 76	0.005
**Onset of PTH** **(days)**	≤7 >7	40 (33.1%) 22 (22.4%)	81 (66.9%) 76 (77.6%)	121 98	0.083
**Blood transfusion**	No Yes	55 (25.7%) 8 (66.7%)	159 (74.3%) 4 (33.3%)	214 12	0.002
**Bleeding source**	One tonsil Both tonsils	42 (28.8%) 15 (44.1%)	104 (71.2%) 19 (55.9%)	146 34	0.083

*Chi-square test

**Fig 2:**
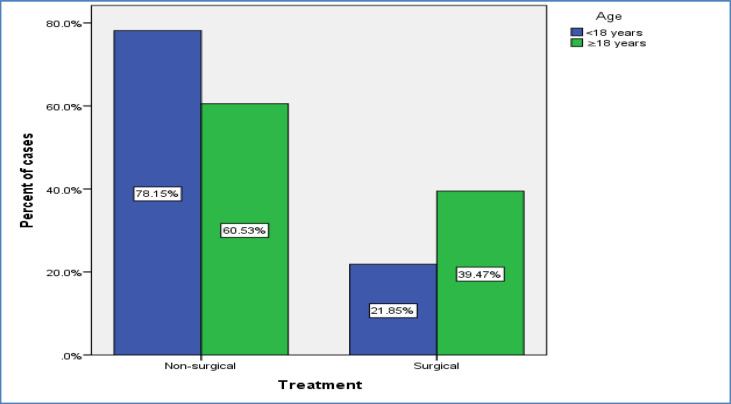
Distribution of cases undergoing intervention and observation in two age groups

As the table implies, there were significant differences between patients undergoing surgical and non-surgical treatment regarding age and receiving blood transfusion (P<0.05). In particular, a significant percentage of adult patients (those aged 18 years or above) required surgical intervention for controlling PTH (P= 0.005, Figure 2). 

Moreover, a significant number of patients who received an intraoperative blood transfusion underwent surgical interventions (P= 0.002).

**Subgroup analyses**

Association of the onset of PTH and the need for surgery to control PTH was assessed in different age groups (Table.2).

**Table 2:** Association of treatment type and time of hemorrhage in different age groups

Age group	Onset of PTH	Treatment		Total	P*
		Surgical	Non-surgical		
**<18 years**	During the first week After the first week	22 (27.2%) 10 (15.6%)	59 (72.8%) 54 (84.4%)	81 64	0.096
**≥18 years**	During the first week After the first week	18 (45.0%) 12 (35.3%)	22 (55%) 22 (64.7%)	40 34	0.397
**≥6 years**	During the first week After the first week	39 (39.4%) 19 (24.4%)	60 (60.6%) 59 (75.6%)	99 78	0.034

*Chi-square test

According to the table, no significant relationship was observed between undergoing surgery and the onset of PTH among the patients under 18 years old, as well as those aged 18 or older (P > 0.05). 

However, a significant association was found between the onset of PTH and treatment type, indicating a significantly higher percent of cases undergoing surgical treatment among the group in whom PTH occurred during the first week after the initial tonsillectomy surgery (P= 0.034).

Multivariate analysis

A multivariate binary logistic regression model showed that among the measured variables, adult age, bleeding from both tonsils, and receiving blood transfusion were independent predictors that significantly increased the possibility of undergoing surgery for controlling PTH (Table.3). 

**Table 3:** The multivariate binary logistic regression model

Variable	Odds ratio	95% confidence interval		P
		Lower bound	Upper bound	
**Gender (Male)**	0.687	0.338	1.398	0.300
**Age (≥18 years)**	4.032	1.932	8.414	<0.001
**Onset of PTH (within first week)**	1.790	0.872	3.673	0.112
**Bleeding source (Both tonsils)**	2.380	1.032	5.487	0.042
**Intraoperative blood transfusion**	7.934	2.003	31.422	0.002


In contrast, gender and time of onset of PTH failed to show a significant relationship with the type of treatment in the multivariate analyses.

## Discussion

Post-tonsillectomy hemorrhage is a major complication of tonsillectomy. This complication rarely happens, but it is important due to the associated complications (8,9). rancis et al., in their systematic review of 87 comparative studies, reported that PTH occurred in < 5% of children. They reported the rate of 0.2% and 2.2% and 0.1% to 3% for primary and secondary PTH, respectively (5, 10). The need for surgery to control PTH was examined in the present study. Overall, 27.8% of the patients underwent surgery to control PTH. The majority of patients experienced PTH within one week after the operation. Significant differences were observed between the patients undergoing surgical and non-surgical treatment regarding age and receiving a blood transfusion. Since most studies report that bleeding is more common in the first week after surgery (11-13), the onset of bleeding was divided into before and after the seventh day in the present study and the relationship between time of bleeding and the probability of surgery was investigated. Generally, there was no association between the onset of PTH and the need for surgical intervention. In the pediatric group, 27.2% of patients who had bleeding before the seventh day required surgery to control bleeding, compared with 15.6% of them with bleeding after the seventh day.

Although the proportion of patients who needed surgery to control PTH was higher during the first seven days in both age groups, this difference was not statistically significant. However, the current study demonstrated that patients aged 6 years or older who had PTH in the first week following the surgery were significantly more likely to need surgical intervention to control bleeding. Therefore, age over 6 years can be considered a factor that might increase the probability of requiring surgical intervention if the bleeding occurs within the first 7 days after surgery. This needs further investigation in studies with larger sample populations. Multivariate regression analyses showed that adulthood, bleeding from both tonsils, and receiving blood transfusions were independent risk factors for surgical treatment. The need for surgery to control PTH was 4 times in adults, while the risk was 2.4 in patients with bleeding from both tonsils and 7.9 in those who received a blood transfusion. However, gender and time of onset of PTH were not independently associated with the need for surgery. PTH often occurs due to the separation of the primary eschar as the tonsil bed heals by secondary intention (2,10). Other etiologies of PTH include tonsillar cellulitis, swallowing solid foods, use of nonsteroidal anti-inflammatory drugs (NSAIDs) after surgery, and idiopathic causes (11,14-17). Several studies have shown that PTH may occur between a day and a month following the initial surgery and is most common during the first 7 days after surgery (2,11-13). This result is consistent with the findings in the present study regarding the most common time of onset of PTH. Susaman et al. stated that PTH occurred in children and adults between days 2 to 15, and 2 to 10, respectively. The most common days were the ninth and tenth days in pediatric cases, and the 5th day in adults (16). Ikoma et al. also reported that PTH usually occurs on days 5 and 6 after the surgery (18). In the present study, the meantime of onset of PTH was about 8 days. Also, the seventh day was the most common day of onset of PTH in adults, while a secondary peak was additionally observed in pediatric cases on day 14. 

On the fifth day after tonsillectomy, the fibrin membrane covers the entire surface of the tonsil bed, and the membrane detaches on the seventh day. Capillary growth is simultaneously at its peak in the first week after surgery. The newly formed vessels are exposed when the fibrin membrane separates and the possibility of bleeding with minor trauma increases. Therefore, it can be concluded that if bleeding occurs in the presence of the fibrin membrane, there is a higher probability of needing an intervention (19).

Most previous studies in line with the present one, have suggested that there is a higher chance of surgery to control the bleeding in older patients with PTH (5,18,20-24). In a prospective multicenter observational study, Tomkinson et al. demonstrated that patients over 12 years of age are more likely to have a bleeding complication than young ones (21). Spektor et al. in their study on a pediatric population found that the risk of PTH increases 1.1 times with each year of age (24). Likewise, Arora et al. in their retrospective review of 181 children with 193 episodes of PTH, concluded that children aged 6 years or older need more surgery than the younger ones (5). Similarly, the present study found that patients over 18 years of age needed more surgery to control bleeding than younger ones.

Several studies that assessed the patients with a history of tonsillectomy have indicated different risk factors such as chronic tonsillitis, excessive bleeding that needs transfusion during the surgery, male, and increased postoperative mean arterial pressure for PTH patients (18,22,24-26). Although the risk factors for the development of PTH were not identified in this study as only patients with PTH were included, the risk factors for the probability of needing surgical control for PTH were assessed. Consistent with the abovementioned studies, intraoperative blood transfusion was considered as an independent predictor of PTH severity, but no significant association was observed between the need for surgery and gender in the present study.

The limitations of this retrospective study were insufficient information that limits assessment and variables such as patient's status cannot be included. Moreover, considering that the ICD-10 code was used to retrieve records, patients who were mistakenly registered using other codes were omitted. Finally, other risk factors such as the chronicity of tonsillitis were not considered in the present study.

## Conclusion

It can be concluded that PTH may require surgery if it occurs within the first postoperative week in patients ≥ 6 years. Additionally, adulthood, bleeding from both tonsils, and receiving blood transfusion were independently associated with the severity of bleeding and could be suggested as potential predictors of the need for surgery in patients with PTH. Therefore, considering these factors and being prepared for surgery to control bleeding is clinically important in patients with PTH.

## Funding

The present study was financially supported by Mashhad University of Medical Sciences [Grant number: 980894].
